# An autoregulatory feedback loop of miR-21/VMP1 is responsible for the abnormal expression of miR-21 in colorectal cancer cells

**DOI:** 10.1038/s41419-020-03265-4

**Published:** 2020-12-14

**Authors:** Caixia Wang, Rui Peng, Min Zeng, Zhenhua Zhang, Shengpeng Liu, Dan Jiang, Yuanyuan Lu, Fangdong Zou

**Affiliations:** 1 College of Life Sciences, Sichuan University, Chengdu, Sichuan P.R. China; 2 West China Hospital of Pathology, Sichuan University, Chengdu, Sichuan P.R. China; 3 Xijing Hospital of Digestive Disease, Fourth Military Medical University, Xi’an, Shanxi P.R. China

**Keywords:** Cell biology, Cancer

## Abstract

MircoRNA-21 (miR-21) was found to be highly expressed in various solid tumors, and its oncogenic properties have been extensively studied in recent years. However, the reason why miR-21 is highly expressed in various tumors remains elusive. Here, we found that the expression of miR-21 was negatively correlated with the expression of vacuole membrane protein-1 (VMP1) in colorectal cancer. Transcription of VMP1 activated either by small activating RNA (saRNA) or transcriptional activator GLI3 inhibited miR-21 expression through reducing its transcripts of VMP1-miR-21 and pri-miR-21, while no significant change in miR-21 expression after exogenous overexpression VMP1 in colorectal cancer cell HCT116. Considering the overlapping location of VMP1 and miR-21 gene in genome, the result suggested that the transcription of miR-21 was inhibited by the endogenous transcriptional activation of VMP1. Furthermore, we identified that miR-21 inhibited the activation and nuclear translocation of transcription factor EB (TFEB) through reducing the inhibitory of PTEN on AKT phosphorylation, which can directly activate the transcription of VMP1. Loss of miR-21 significantly increased VMP1 expression, which could be blocked by PTEN inhibitor (VO-Ohpic) or TFEB siRNA. These results showed that miR-21 negatively regulated VMP1 transcription through the PTEN/AKT/TFEB pathway, and TFEB-induced transcriptional activation of VMP1 could inhibit miR-21 expression, thus forming a feedback regulatory loop of miR-21/VMP1. We further found that disrupting the miR-21/VMP1 feedback loop will decrease the expression of miR-21, reduce the malignancy, and increase their sensitivity to 5-fluorouracil in colorectal cancer cells. Taken together, our results revealed a novel regulatory mechanism of miR-21 expression, and targeting the miR-21/VMP1 feedback loop may provide a new approach to inhibit miR-21 expression in colorectal cancer cells.

## Introduction

MicroRNAs are short endogenous non-coding RNAs that post-transcriptionally regulate the expression of their target genes. The biological regulation of microRNAs is an important part of cell life activities. Dysregulation of microRNAs expression will lead to developmental defects and a variety of human diseases. A lot of studies have shown that miR-21 is abnormally highly expressed in various tumors, which is considered as a marker of malignant tumors and poor prognosis^1–5^. miR-21 exerts its oncogenic role mainly through the post-transcriptionally regulation of its target genes, such as cell division cycle 25 A (CDC25A), programmed cell death protein 4 (PDCD4), phosphatase and tensin homolog (PTEN), tropomyosin 1 (TPM1), and sprouty RTK signaling antagonist 2 (SPRY2)^6–10^, suggesting that miR-21 could function in cell proliferation, apoptosis, invasion, metastasis, and drug resistance. The oncogenic properties of miR-21 have been extensively studied in recent years. It is known that transcription factors, such as STAT3, Bcl6, AP-1, IL6, and p53, directly regulate miR-21 expression^11–14^. In addition, a double-negative feedback loop regulatory mechanism of miR-21 and AP-1 has been demonstrated^13^. Importantly, the miR-21/AP-1 feedback loop is highly activated during tumorigenesis, and miR-21 maintained itself at constant high levels via this autoregulatory feedback loop, which may also be one of the reasons for the abnormal high expression of miR-21 in tumors^15,16^. However, the reasons why miR-21 is highly expressed in tumors is still largely not understood.

miR-21 is located on chromosome 17q23.2, close to the downstream of Vacuole membrane protein-1(VMP1). A previous study has identified a fusion transcript of VMP1-miR-21 in multiple cell types^17^, indicating that the transcription of miR-21 is regulated not only by its own promoter, miPPR, but also by the VMP1 promoter. In recent years, the biological function of VMP1 has been increasingly elucidated. It has been demonstrated that VMP1 can promote the formation of autophagosomes by regulating the interaction between endoplasmic reticulum (ER) and isolated membrane when VMP1 is located on ER membrane^18–20^. Furthermore, VMP1 is essential for the formation of initial cell-cell contacts and tight junction, and its expression level is associated with the invasion and metastatic potential of cancer cells. The expression of VMP1 in colorectal cancer (CRC) tissues was lower than that in adjacent non-cancer tissues, showing a negative correlation with the malignancy of the cancer^21^. Downregulation of VMP1 results in loss of cell adherence and increase of the invasion capacity^22,23^.

In this study, we identified a novel feedback loop regulating miR-21 expression, in which miR-21 inhibits the expression of VMP1 through the PTEN/TFEB pathway in CRC cells, which in turn directly negatively regulates miR-21 expression by transcriptional repression, thus forming a feedback loop, miR-21/VMP1. Due to the important role of VMP1 and miR-21 in the development of tumor, this novel regulatory mechanism will contribute to explore the tough issues in CRC, such as proliferation, invasion, metastasis, and drug resistance.

## Results

### The expression of miR-21 is negatively correlated with VMP1 expression in CRC

**Fig. 1:**
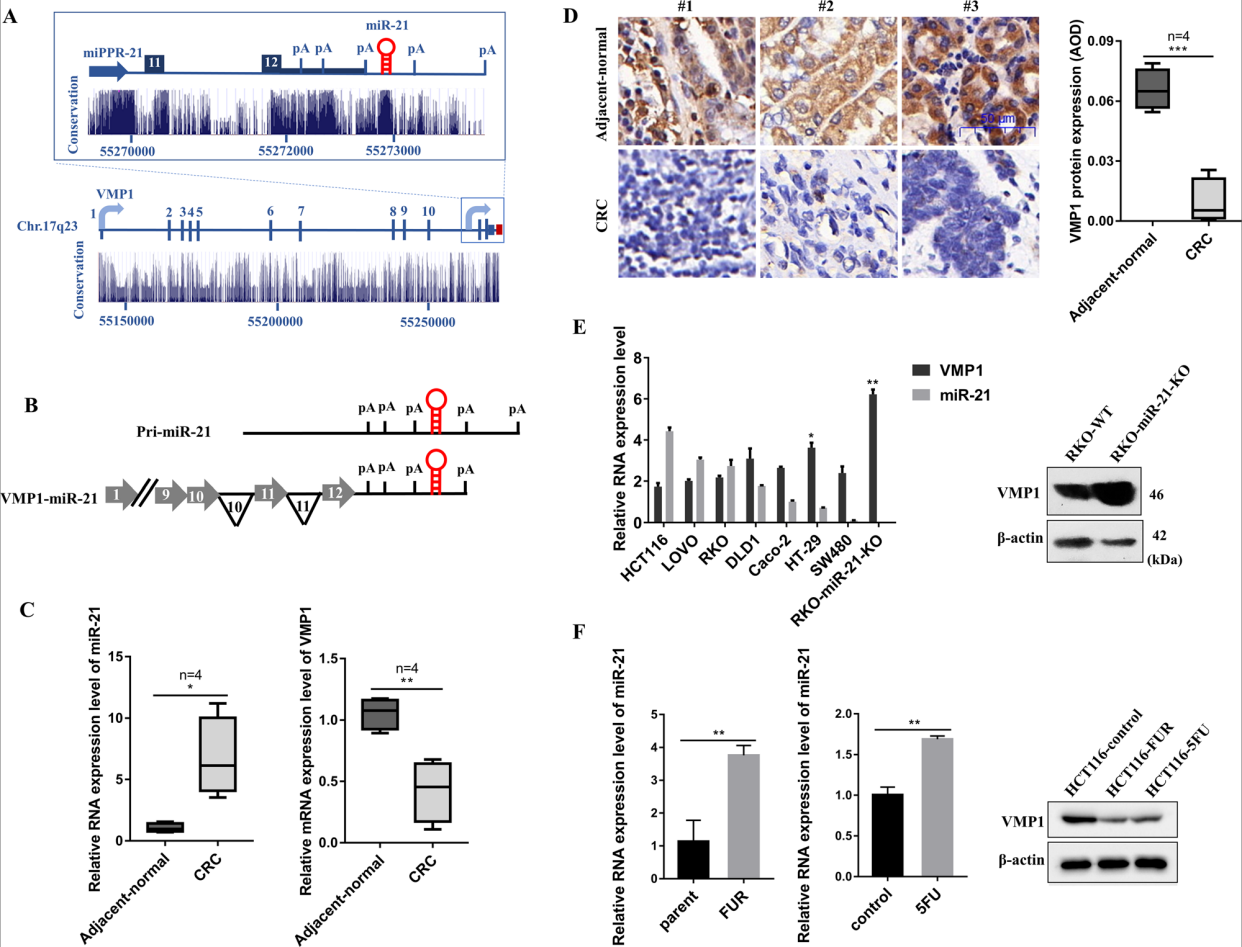
The expression of miR-21 is negatively correlated with VMP1 in colorectal cancer. **a** Structure of the VMP1 and miR-21 loci at chromosome 17q23. VMP1 exon numbers are noted, inset highlights the pri-miR-21 gene region. The blue-black arrow is situated at the miR-21 promoter, miPPR-21. The line of intermediate thickness indicates the 3-UTR of VMP1. The red hairpin indicates the location of the pre-miR-21 hairpin. Polyadenylation signals are indicated as pA. Evolutionary conservation (UCSC Genome Browser 28 species conservation track, NCBI36/hg18 assembly) is plotted below. **b** Schematic of the transcripts pri-miR-21 and VMP1-miR-21. Horizontal gray arrows represent exons and black lines represent introns (numbered in black) and non-coding sequences. Break symbols (double slash mark) are placed between exons 1 and 9 of VMP1. **c** qPCR analysis for miR-21 and VMP1 expression in colorectal cancer tissues and adjacent non-cancer tissues. **d** IHC analysis for the protein expression level of VMP1 in colorectal cancer tissues and adjacent non-cancer tissues. **e** The mRNA expression level of miR-21 and VMP1 was measured by qPCR in various colorectal cancer cells. The protein expression of VMP1 was measured by Western blot in parental RKO and RKO-miR-21-KO cells. **f** The mRNA expression of miR-21 was measured by qPCR, the protein expression of VMP1 was measured by Western blot in HCT116 cells treated with 10 µM 5-FU for 24 h and HCT116-5FUR cells, compared with the control group. (Scale bars, 50 µm) Data are presented as mean ± SD. ***P < 0.001, **P < 0.01, *P < 0.05.

Many studies have revealed that miR-21 was abnormally highly expressed in a variety of tumors, and exerted as an “oncomiR”. miR-21 gene is located in the 10th intron of the VMP1 gene in the human genome. Except for the transcript of pri-miR-21, a fusion transcript of VMP1-miR-21 has been identified (Fig. 1a, b). we used Oncomine datasets to analyze the expression of miR-21 and VMP1 in CRC tissues, and found that compared with human normal colorectal tissues, miR-21 mRNA levels were significantly increased in CRC tissues, conversely, VMP1 mRNA levels were significantly decreased in CRC tissues (Fig. S1A). Moreover, we examined the expression of miR-21 and VMP1 in human CRC tissues and paired adjacent normal tissues by qPCR and immunohistochemistry (IHC). As shown in Fig. 1c, d, miR-21 was a higher expression and VMP1 was a lower expression in CRC tissues compared with the adjacent normal tissues. Therefore, we speculated that there may be a relationship between the VMP1 and miR-21 expression in CRC. To test this hypothesis, we detected the expression of miR-21 and VMP1 in different CRC cells, and found that miR-21 expression was negatively correlated with the expression of VMP1, the expression of VMP1 in miR-21 deficient cells (RKO-miR-21-KO) was much higher than that in parental RKO cells (Fig. 1e).

5-Fluorouracil (5-FU) is a classic chemotherapeutic drug that has been widely used for CRC treatment^24,25^. It is known that 5-FU can increase miR-21 expression in CRC cells^26^. To further investigate the relationship between miR-21 and VMP1 expression, we firstly developed a 5-FU resistant cell line, HCT116-FUR, and the concentration of 5-FU resistance increased eightfold in HCT116-FUR cells, compared to that in parental HCT116 cells (Fig. S1B). As shown in Fig. 1f, the expression of miR-21 and VMP1 in HCT116-FUR showed a similar trend in HCT116 cells with the treatment of 5-FU. miR-21 was significantly upregulated, while VMP1 was down-regulated compared with the parental HCT116 cells. These results indicate that the expression of miR-21 is negatively correlated with VMP1 expression in CRC cells.

### Transcriptional activation of VMP1 inhibits miR-21 expression

**Fig. 2:**
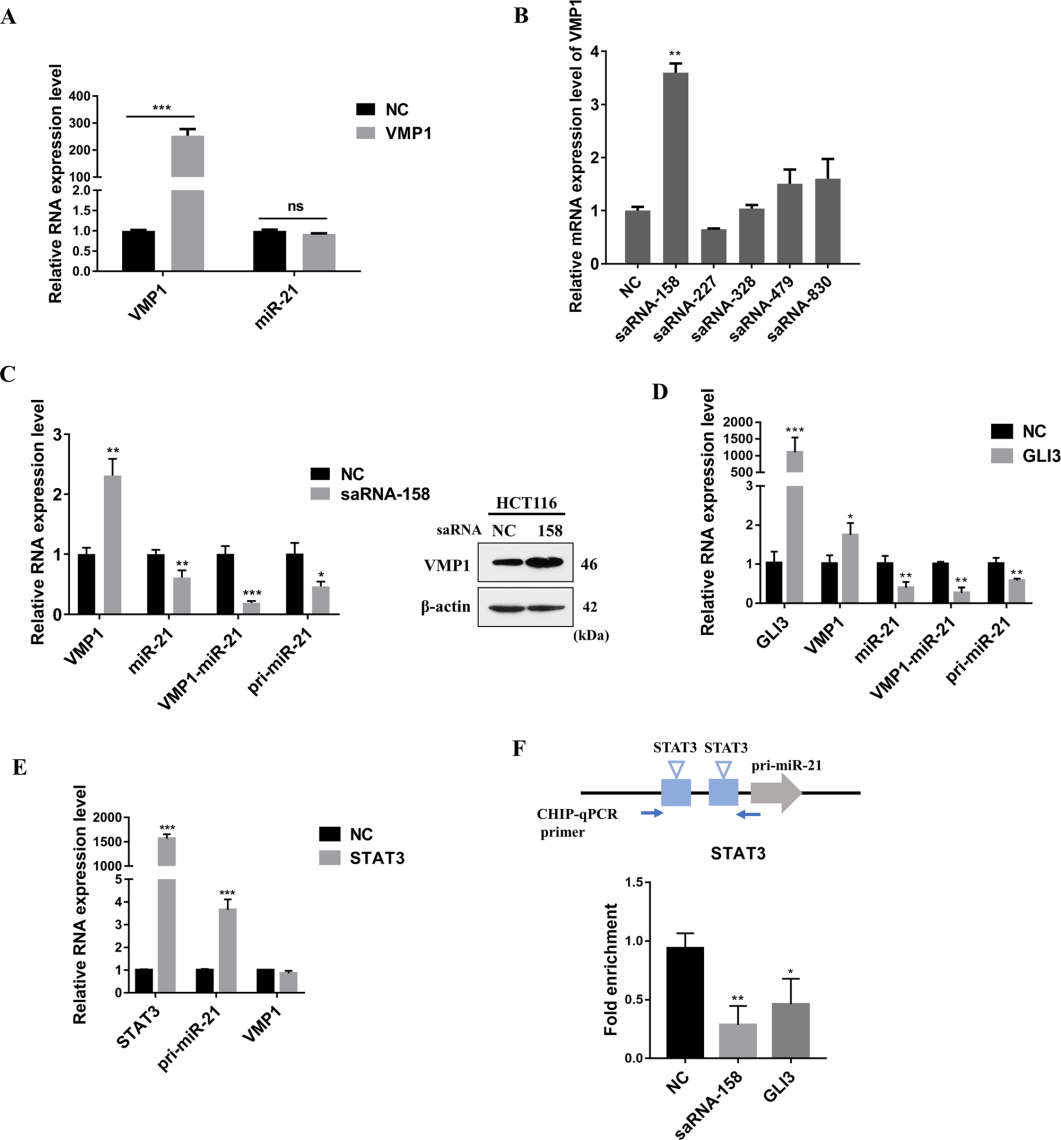
Transcriptional activation of VMP1 inhibits miR-21 expression. **a** HCT116 was transfected with pcDNA3.1-VMP1 vector for 48 h, the mRNA expression level of VMP1 and miR-21 was measured by qPCR. **b** HCT116 was transfected with the small activating RNA (saRNA) targeting VMP1 promoter for 48 h, then the mRNA expression of VMP1 was measured by qPCR. **c** HCT116 was transfected with saRNA-158 for 48 h, the mRNA and protein expression level of VMP1 was measured by Western blot and qPCR, miR-21 expression and the number of pri-miR-21 and VMP1-miR-21 transcripts were measured by qPCR. **d** HCT116 was transfected with pEGFP-C3-hGLI3 vector for 48 h, the mRNA expression level of GLI3, VMP1, miR-21, and the number of pri-miR-21 and VMP1-miR-21 transcripts were measured by qPCR. **e** HCT116 was transfected with pEGFP-N1-STAT3 vector for 48 h, the mRNA expression level of STAT3, VMP1, and the number of pri-miR-21 were measured by qPCR. **f** HCT116 was transfected with saRNA-158 and pEGFP-C3-hGLI3 vector, then CHIP-qPCR analyses using phospho-STAT3 (Tyr705) special antibody were performed. Primer specific for the binding sites of STAT3 to the pri-miR-21 promoter region. Data are presented as mean ± SD. ***P < 0.001, **P < 0.01, *P < 0.05.

Due to that VMP1 is a critical membrane protein involved in autophagosome formation. We increased VMP1 expression through glucose starvation or rapamycin treatment, and found that the expression of miR-21, both pri-miR-21 and VMP1-miR-21 transcripts, were decreased (Fig. S2B, C). The different primers for qPCR of VMP1, VMP1-miR-21 and pri-miR-21, miR-21 were shown in Fig. S2A. To explore whether VMP1 regulates miR-21 expression, we first overexpressed exogenous VMP1 in HCT116 cells and found no significant change in miR-21 expression (Fig. 2a), suggesting VMP1 did not affect the expression of miR-21 through its protein level. To further explore whether the transcription of VMP1 represses miR-21 transcription, Small activating RNAs (saRNAs) are sized 21-nt dsRNAs targeting the promotor region of the target gene to cause a long-lasting and specific induction^27^. Thus, five saRNAs targeting the promoter region of the VMP1 gene were synthesized to enhance its transcription. The efficiency of saRNAs was determined by detecting the mRNA level of VMP1. Of these five saRNAs, only the saRNA targeting the upstream 158 bp of VMP1 promotor region significantly increased the expression of VMP1 (Fig. 2b). As shown in Fig. 2c, both miR-21 and its transcripts of VMP1-miR-21 and pri-miR-21 were down-regulated after saRNA-158 activated the transcription of VMP1. In addition, GLI3, a known transcription factor of VMP1^28^, also showed a similar effect to saRNA-158 in the regulation of miR-21 transcription (Fig. 2d).

Transcriptional interference (TI) has been broadly found in many species and defined as the direct negatively impact of one gene’s transcription on the second neighboring gene that is located in cis^29^. The transcriptional elongation of upstream gene across the promoter region of the downstream gene may directly block the binding of transcription factors of the downstream gene^30^. Considering the promoter region of miR-21 gene was located in the coding region of VMP1, we speculated that transcriptional activation of VMP1 decreased pri-miR-21 transcript through TI. To test this hypothesis, we employed CHIP assay to detect the binding change of STAT3, a known transcription factor of pri-miR-21, on pri-miR-21 promoter after activation of VMP1 transcription. As expected, STAT3 significantly promote pri-miR-21 transcription (Fig. 2e), and CHIP result showed that the binding of STAT3 to the pri-miR-21 promoter is significantly reduced when VMP1 transcription was activated by saRNA-158 or GLI3 (Fig. 2f). Together, these results indicate that transcriptional activation of VMP1 inhibits miR-21 expression.

### miR-21 inhibits VMP1 expression through PTEN/AKT/TFEB pathway

**Fig. 3:**
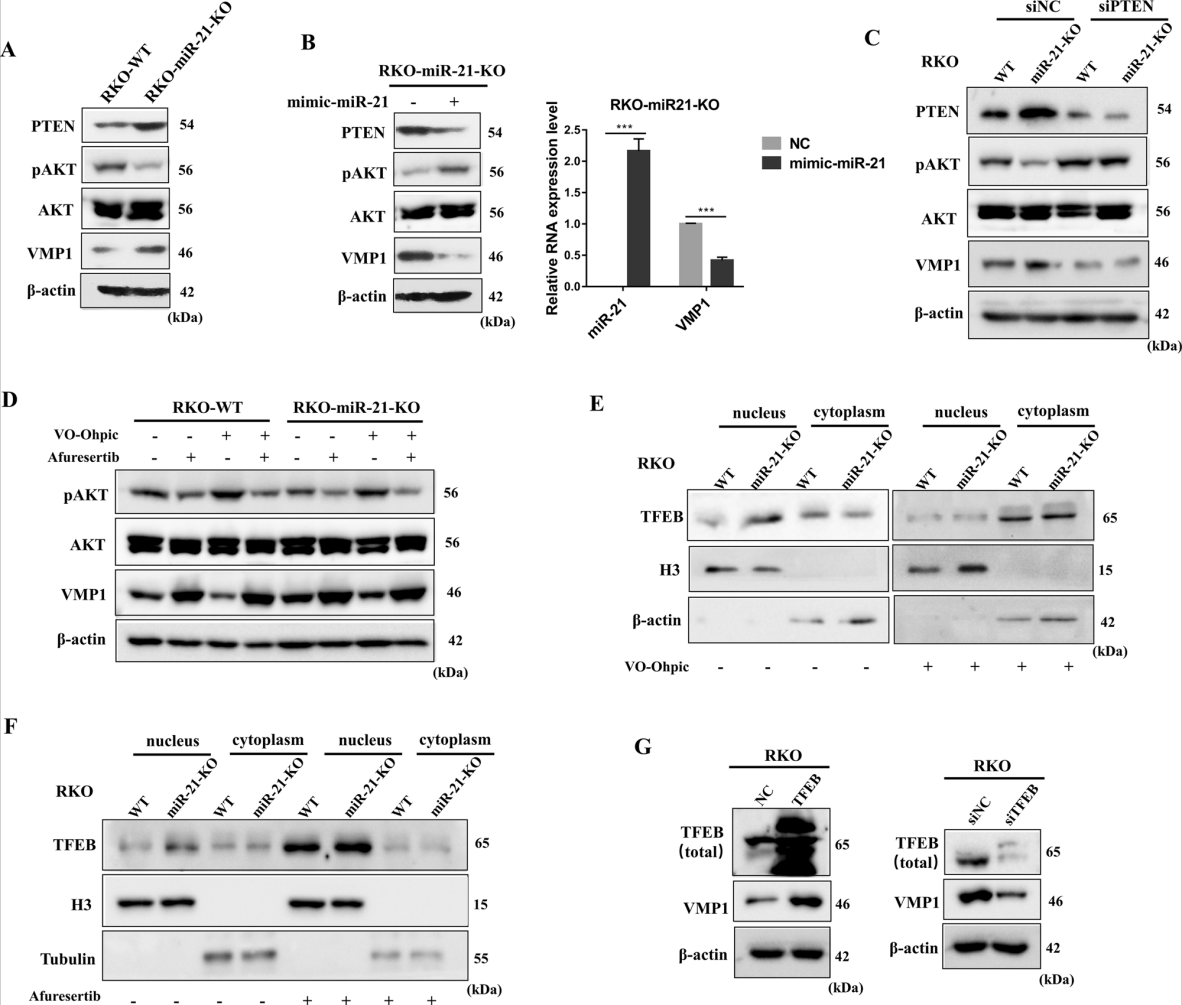
miR-21 inhibits VMP1 expression through PTEN/AKT/TFEB pathway. **a** The protein expression level of PTEN, phosphorylated AKT (Ser473), total AKT, and VMP1 were measured by Western blot. **b** RKO-miR-21-KO was transfected with miR-21 nucleotide mimic, and the mRNA expression level of miR-21 and VMP1 was measured by qPCR, the protein expression level of PTEN, phosphorylated AKT (Ser473), total AKT, and VMP1 were measured by Western blot. **c** RKO and RKO-miR-21-KO were transfected with siRNA targeting PTEN respectively, the protein expression level of PTEN, phosphorylated AKT (Ser473), total AKT, and VMP1 were measured by Western blot. **d** RKO and RKO-miR-21-KO were treated with 2 µM VO-Ohpic and 1 µM Afuresertib for 24 h, the protein expression level of phosphorylated AKT (Ser473), total AKT, and VMP1 in after were measured by Western blot. **e** RKO and RKO-miR-21-KO cells with no treat or treat with 2 µM VO-Ohpic for 24 h, the protein level of TFEB in cytoplasm and nucleus respectively was measured by Western blot, H3 and β-actin were used as nuclear and cytosolic markers, respectively. **f** RKO and RKO-miR-21-KO cells with no treat or treat with 1 µM Afuresertib for 24 h, the protein level of TFEB in cytoplasm and nucleus respectively was measured by Western blot, H3 and Tubulin were used as nuclear and cytosolic markers, respectively. **g** The protein expression level of TFEB and VMP1 were measured by Western blot after overexpression of the constitutively active TFEB by pCIP-caTfeb vector or knock-down of TFEB expression by siRNA in RKO cells. Data are presented as mean ± SD. ***P < 0.001.

Since knock-out of miR-21 increased VMP1 expression (Figs. 1e, 3a), we therefore explored whether miR-21 negatively regulates VMP1 expression. As expected, the rescue of the miR-21 expression by miR-21 nucleotide mimics in RKO-miR-21-KO cells, significantly decreased the expression of VMP1(Fig. 3b), indicating that miR-21 inhibits VMP1 expression. Using miRTarBase and TargetScan datasets, we found that there was no miR-21 binding site on the VMP1 mRNA sequence.

Next, we explored the molecular mechanism by which miR-21 inhibits VMP1 expression. It is known that PTEN is a direct target gene of miR-21 and functions as an upstream inhibitor of AKT activation, and it has been proven that miR-21 increased AKT phosphorylation by inhibiting the expression of PTEN in CRC cells^31^. Similar to the reported study, the expression of PTEN was upregulated, while phosphorylated AKT (Ser473) was down-regulated in RKO-miR-21-KO cells compared with parental RKO cells, and their expression showed an opposite trend after the rescue of miR-21 expression in RKO-miR-21-KO cells (Fig. 3a, b). Therefore, we speculated that PTEN/AKT probably involved in the regulation of miR-21 on VMP1 expression. To address this issue, we first silenced PTEN expression by siRNA in RKO and RKO-miR-21-KO cells, and the result showed that knock-down of PTEN expression increased AKT phosphorylation and decreased the expression of VMP1, especially in RKO-miR-21-KO cells (Fig. 3c). Meanwhile, we found that the expression of VMP1 was inhibited by the PTEN inhibitor VO-Ohpic, promoted by the AKT inhibitor Afuresertib, and VO-Ohpic-induced downregulation of VMP1 expression was completely rescued by Afuresertib (Fig. 3d). Suggesting that miR-21 inhibits VMP1 expression through the PTEN/AKT axis.

AKT has been reported to directly phosphorylate Transcription factor EB (TFEB) at Ser467 and repressed its nuclear translocation, and TFEB activity is critical for the expression of autophagy-related genes^32^. In this study, we found that the nuclear TFEB in RKO-miR-21-KO was higher than that in parental RKO cells, and the accumulation of TFEB in the nucleus of RKO-miR-21-KO cells was blocked by PTEN inhibitor, VO-Ohpic (Fig. 3e). While the AKT inhibitor, Afuresertib, enhanced nuclear translocation of TFEB in both RKO and RKO-KO cells (Fig. 3f). Meanwhile, Afuresertib can rescue the nuclear translocation of TFEB inhibited by miR-21 overexpression (Fig. S3A). These results indicate that miR-21 inhibits the nuclear translocation of TFEB via PTEN/AKT. In addition, we found that the expression of VMP1 was concomitantly increased or decreased in response to overexpression of the constitutively active TFEB or knock-down of TFEB expression by siRNA in RKO and HCT116 cells (Fig. 3g and Fig. S3B), suggesting that TFEB promotes VMP1 expression. The upregulation of VMP1 induced by miR-21 deficiency was inhibited by TFEB siRNA (Fig. 5f). Taken together, our study demonstrated that miR-21 inhibits VMP1 expression through the PTEN/AKT/TFEB axis.

### TFEB regulates VMP1 transcription by directly binding to the promoter region of VMP1

**Fig. 4:**
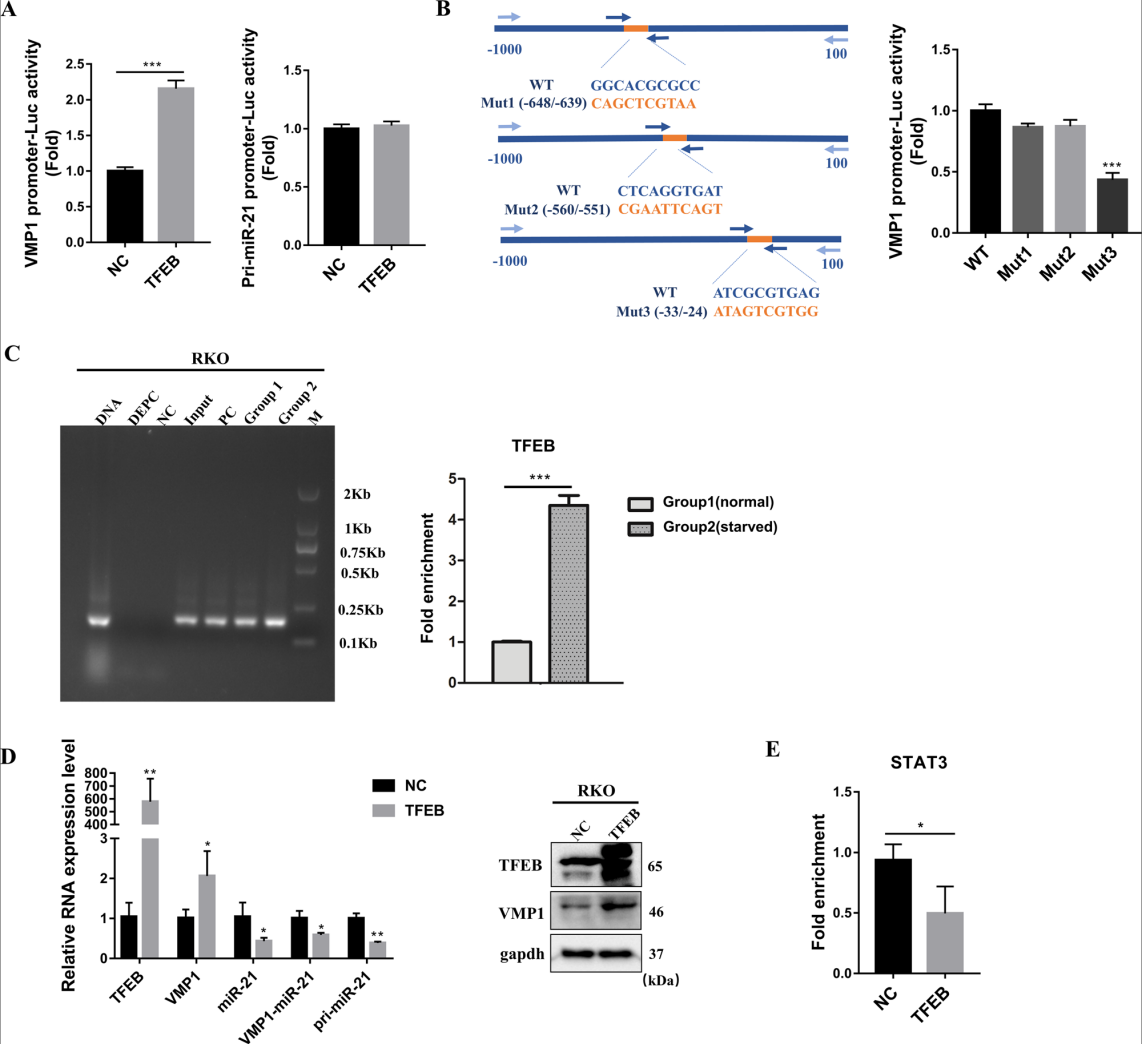
TFEB regulates VMP1 transcription by directly binding to the promoter region of VMP1. **a** RKO was transfected with pCIP-caTfeb vector and the construct dual-luciferase vectors, pmiRGLO-VMP1 promoter (−1000/+100) or pmiRGLO-pri-miR-21 promoter (−1000/+100), the luciferase activity was measured. **b** Luciferase report assay using the construct vector carrying the VMP1 promoter region, in which the predicted most likely three binding site (−648/−639, −560/−551, −33/−24, the upstream of the transcription start site of VMP1) with TFEB was mutant. **c** DNA Agar-gel electrophoresis from CHIP-qPCR assay, the amount of immunoprecipitated DNA isolated from TFEB special antibody/protein G magnetic beads was measured by qPCR. Primer specific for VMP1 promoter region (−116/+27). NC: negative control, PC: positive control, Group1: cells with no treat, Group2: cells with glucose starvation. **d** The mRNA expression level of TFEB, VMP1, miR-21 and the number of pri-miR-21 and VMP1-miR-21 transcripts were measured by qPCR, the protein expression level of TFEB and VMP1 were measured by Western blot after overexpression of the constitutively active TFEB by pCIP-caTfeb vector in RKO cells. **e** RKO was transfected with pCIP-caTfeb vector, then CHIP-qPCR analyses using phospho-STAT3 (Tyr705) special antibody were performed. Data are presented as mean ± SD. ***P < 0.001, **P < 0.01, *P < 0.05.

Since TFEB, an important transcription factor for the formation of autophagosome, promoted VMP1 expression (Fig. 3g), we therefore investigated whether TFEB directly regulates VMP1 as a transcription factor. To confirm this, we transfected pCIP-caTfeb and the constructed pmiRGLO-VMP1 promoter (−1000/+100) vector into RKO cells, the result showed that TFEB increased the activity of VMP1 promoter-Luc, and had no effect on the activity of pri-miR-21 promoter-Luc (Fig. 4a), suggesting that TFEB can promote VMP1 transcription but has no effect on pri-miR-21 transcription. To further clarify the binding site of TFEB on the VMP1 promoter, we mutated the three predicted binding sites which were predicted from the JASPAR CORE database in the −1000/+100 promoter region of VMP1 and generated the dual-Luciferase reporter constructs. As shown in Fig. 4b, mutating the −33/−24 region significantly reduced the transcriptional activity of TFEB, but mutant with −560/−551 region and −648/−639 region had no similar effect. Moreover, the result of CHIP also revealed that TFEB can specifically bind to the region between −116 and +27 of VMP1 promoter, and glucose starvation increased the binding of TFEB to the VMP1 promoter (Fig. 4c), suggesting that TFEB could regulate VMP1 transcription by directly binding to the −33/−24 region of VMP1 promoter.

In keeping with the results in Fig. 2c, d, the transcriptional activation of VMP1 by TFEB down-regulated the expression of mature miR-21, VMP1-miR-21, and pri-miR-21 transcript (Fig. 4d). Furthermore, overexpression of TFEB also repressed the binding of the transcription factor STAT3 to pri-miR-21 promoter (Fig. 4e). Together, our study demonstrated that miR-21 inhibits VMP1 expression through PTEN/AKT/TFEB axis, and TFEB-induced transcriptional activation of VMP1 could inhibit miR-21 expression, thus forming a feedback regulatory loop of miR-21/VMP1.

### miR-21 inhibits the autophagy of CRC cells through PTEN/AKT/TFEB

**Fig. 5:**
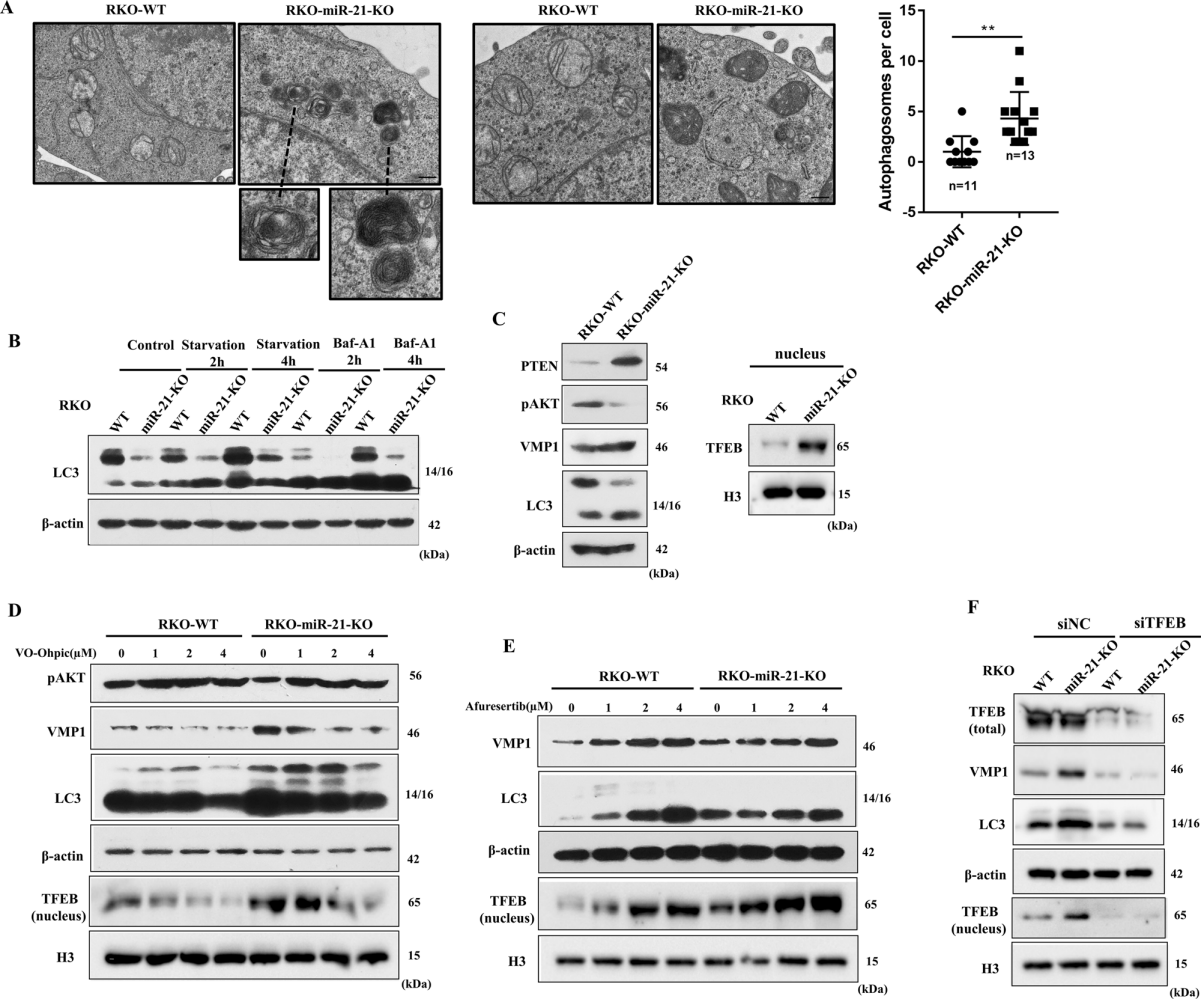
miR-21 regulates the autophagy of colorectal cancer cells through PTEN/AKT/TFEB. **a** Autophagic structures (AV, AL, and IM) were observed in parental RKO and RKO-miR-21-KO cells by transmission electron microscope. Number of autophagic structures was counted. **b** RKO and RKO-miR-21-KO were treated with glucose starvation or bafilomycin A1, the LC3 (LC3I and LC3II) expression was measured by Western blot. **c** The protein expression level of PTEN, phosphorylated AKT (Ser473), VMP1, LC3, and nuclear TFEB were measured by Western blot in RKO and RKO-miR-21-KO cells. **d** RKO and RKO-miR-21-KO were treated with VO-Ohpic at concentrations of 1–4µM for 24 h, and immunoblot analysis of phosphorylated AKT (Ser473), VMP1, LC3 expression and the protein amount of nuclear TFEB. **e** RKO and RKO-miR-21-KO were treated with Afuresertib at concentrations of 1–4 µM for 24 h, and immunoblot analysis of VMP1, LC3 expression, and the protein amount of nuclear TFEB. **f** The protein expression level of VMP1, LC3, total TFEB, and the protein amount of nuclear TFEB were measured by Western blot after knock-down of TFEB expression by siRNA in RKO and RKO-miR-21-KO cells. (Scale bars, 0.5 µm) Data are presented as mean ± SD. **P < 0.01.

Considering the role of TFEB and VMP1 in regulating autophagy, we further investigate whether miR-21 regulates the autophagy in CRC cells through TFEB or VMP1. Under an electron microscope, there were more autophagic structures in RKO-miR-21-KO cells than that in parental RKO cells (Fig. 5a). Meanwhile, LC3II/I was increased in RKO-miR-21-KO cells compared to parental RKO cells under normal condition or treatment with glucose starvation and lysosomal inhibitor bafilomycin A1 (Fig. 5b), indicating that miR-21 inhibits autophagy occurrence. Since the expression of PTEN, nuclear TFEB, and autophagic genes VMP1, LC3II/I were increased, while AKT phosphorylation was decreased in RKO-miR-21-KO compared with parental RKO cells. We then treated RKO and RKO-miR-21-KO cells with PTEN inhibitor VO-Ohpic and AKT inhibitor Afuresertib, the result showed that miR-21 inhibited the nuclear translocation of TFEB and the expression of autophagic genes VMP1, LC3 through PTEN/AKT (Fig. 5d, e). Knock-down of TFEB blocked the inhibitory of miR-21 on VMP1 and LC3 expression (Fig. 5f). Together, these data indicated that miR-21 inhibited cell autophagy through the PTEN/AKT/TFEB pathway.

### Breaking the feedback loop of miR-21/VMP1 reduces the malignancy of CRC cells

**Fig. 6:**
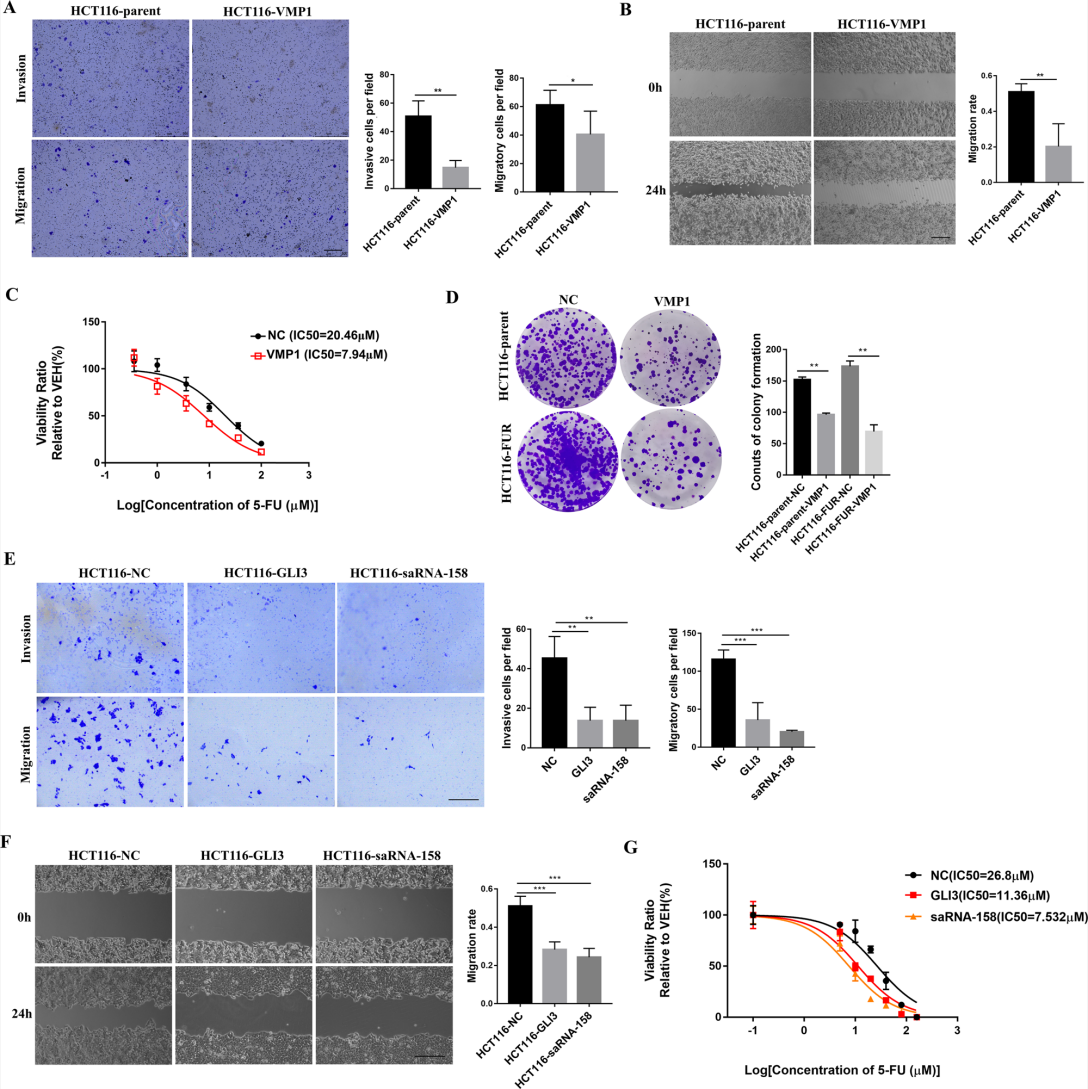
Breaking the feedback loop of miR-21/VMP1 reduces the malignancy of colon cancer cells. **a**, **b** HCT116 with stable overexpression of VMP1 was developed, then the capacity of cell invasion and migration was detected by Tranwell and Wound Healing. **c** HCT116 was transfected with pcDNA3.1-VMP1 vector, and the cell survival rate to 5-FU was measured by MTT assay. **d** HCT116 and HCT116-5FUR cells were transfected with pcDNA3.1-VMP1 vector, then 800 cells were seed in the 6-well plate, and cultured for 2 weeks. Number of colonies was measured. **e**, **f** HCT116 was transfected with saRNA-158 and pEGFP-C3-hGLI3 vector, then the capacity of cell invasion and migration was detected by Tranwell and Wound Healing. **g** HCT116 was transfected with saRNA-158 and pEGFP-C3-hGLI3 vector, and the cell survival rate to 5-FU was measured by MTT assay. (Scale bars, 0.5 mm) Data are presented as mean ± SD. ***P < 0.001, **P < 0.01, *P < 0.05.

As reported, VMP1 protein not only affects the occurrence of autophagy, but also is involved in the proliferation and metastatic potential of cancer cells^19,22,23^. We found that overexpression of VMP1 not only inhibited the invasion and metastasis of HCT116 cells (Fig. 6a, b), but also increased their sensitivity to chemotherapeutic drug, 5-FU (Fig. 6c). In addition, VMP1 inhibited colony formation in both parental HCT116 and HCT116-FUR cells (Fig. 6d), suggesting overexpression of VMP1 inhibited the metastasis, proliferation, and drug resistance in CRC cells.

In this study, we have found that highly expressed miR-21 inhibits the transcription of VMP1, which in turn enhances the expression of miR-21. Considering the role of miR-21 and VMP1 in tumorigenesis, we speculated that the miR-21/VMP1 feedback loop may be one of the reasons for the high expression of miR-21 in tumors. To test this hypothesis, we employed GLI3 or saRNA to activate VMP1 transcription, thus blocking the miR-21/VMP1 feedback loop. The results showed that breaking the feedback loop of miR-21/VMP1 not only significantly reduced the migration and invasion of HCT116 cells (Fig. 6e, f), but also increased their sensitivity to 5-FU (Fig. 6g). Taken together, the results indicated that blocking the feedback regulatory loop of miR-21/VMP1 could increase VMP1 expression and reduce miR-21 expression, resulting in reduced malignancy of CRC cells.

## Discussion

**Fig. 7:**
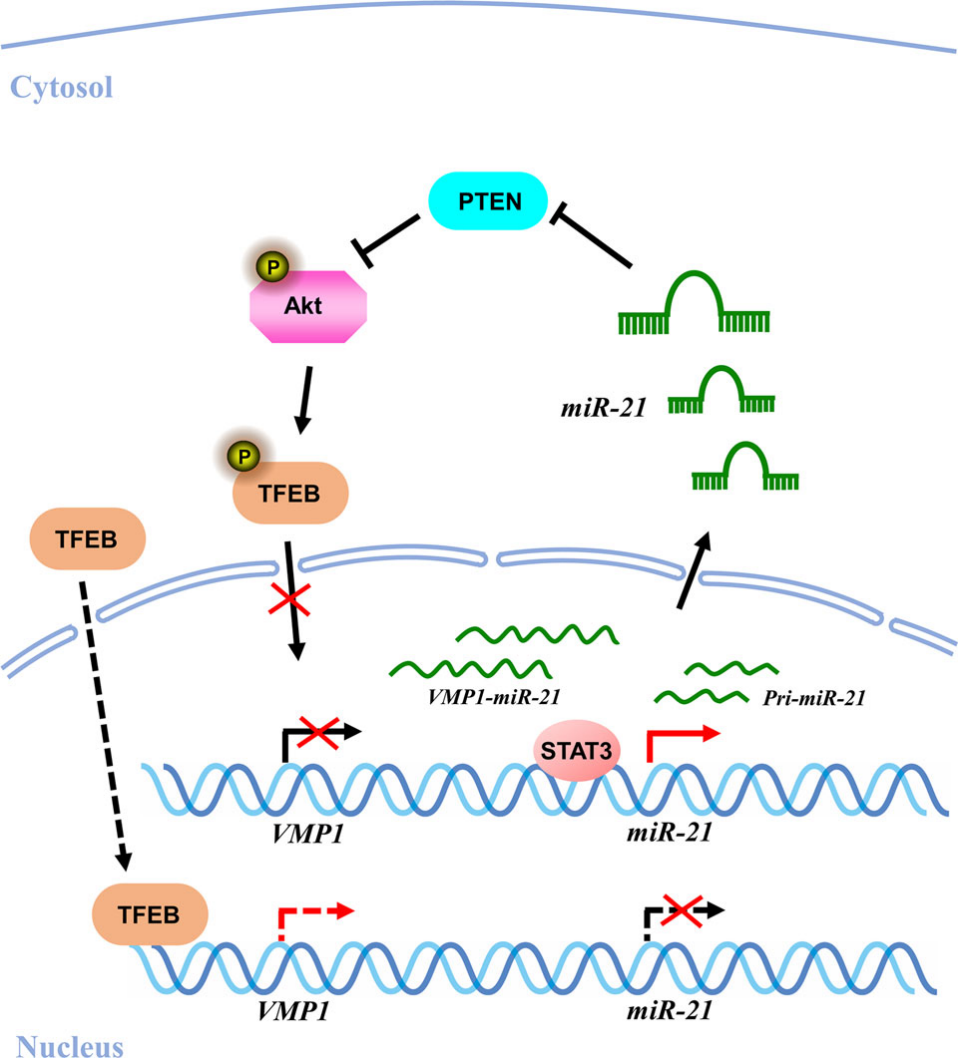
Proposed model for VMP1 medicated the new feedback regulation mechanism of miR-21 via PTEN/AKT/TFEB pathway in colorectal cancer. When TFEB was activated and transported into the nuclear in cells, VMP1 transcript was upregulated and VMP1-miR-21 transcript was down-regulated, Meanwhile, pri-miR-21 transcript was also reduced due to the binding of transcription factors to pri-miR-21 promoter were blocked by TFEB-induced high transcriptional activation of VMP1, such as STAT3 (dashed lines). When VMP1 transcription was inhibited by TFEB phosphorylation, both VMP1-miR-21 and pri-miR-21 transcripts were increased. Subsequently, the high expression of miR-21 increased phosphorylation of AKT by inhibiting PTEN, and then phosphorylated AKT promoted phosphorylation of TFEB and repressed its nuclear translocation, leading to VMP1 transcription was further inhibited and miR-21 transcription was further increased (solid lines).

miR-21 plays a crucial role in many biological processes and diseases by downregulating its target gene expression^14^. Although there were few reports about the regulation of miR-21 expression in recent years^33,34^, the exact mechanism that how miR-21 is upregulated in tumors is still unclear. In this study, we found a novel regulatory mechanism of miR-21, the feedback loop of miR-21/VMP1 (Fig. 7). The expression of miR-21 is negatively correlated with VMP1 in CRC cells, and VMP1 inhibits miR-21 expression at the transcriptional level because of their special locations in genome. On the one hand, transcriptional activation of VMP1 reduced VMP1-miR-21 transcript probably due to the alter-native polyadenylation of VMP1. On the other hand, transcriptional activation of VMP1 reduced pri-miR-21 transcript by interfering the binding of the transcription factor STAT3 to the pri-miR-21 promoter. Both of VMP1-miR-21 and pri-miR-21 as the substrates of the Microprocessor complex to produce mature miR-21, thus the downregulation of these two transcripts could result in lower expression of mature miR-21. Moreover, our studies have also shown that miR-21 can also negatively regulate VMP1 expression through the PTEN/AKT/TFEB pathway. Thus miR-21 could increase itself expression via this feedback regulatory loop of miR-21/VMP1. A similar feedback loop regulatory mechanism, miR-21/AP-1, has been reported in hepatoma and renal carcinoma^13,15,16^. miR-21 promoted AP1 expression through PDCD4, in turn, AP1 directly promotes miR-21 transcription as a transcription factor of miR-21^13^, by which miR-21 maintained itself expression at a constant high level, and functioned as a driving force in tumorigenesis. In addition, the double-negative feedback regulation loop, miR-21/NFIB/miR-21, has been found as an approach to sustain miR-21 expression in HL60 cells^35^, suggesting that the feedback regulation is one of the main regulatory mechanisms of miR-21. Targeting the miR-21/VMP1 feedback loop may provide a new approach to inhibit miR-21 expression in CRC cells.

A previous study has reported that VMP1 and pri-miR-21 were induced by PMA and androgens stimulation while epigenetic modifying agents had different effects on their transcripts^17^. Here, we revealed that the expression of miR-21 is negatively correlated with VMP1 in CRC cells. It’s known that miR-21 expression is induced by PMA or androgens through enhancing the interaction of AP-1 or AR on the pri-miR-21 promoter^35,36^. In this study, we used the JASPAR CORE database to analyze the transcription factor binding sites and found that there are both of several binding sites of AP-1 and AR within the −1000/+100 promoter region of VMP1 (Fig. S3C). Therefore, AP-1 and androgens may directly induce the expression of both VMP1 and miR-21, and the inhibitory effect between miR-21 and VMP1 would likely be mild in this case. In addition, the differential responses to epigenetic modifying agents are probably due to the differential epigenetic modification of VMP1 and miR-21 gene regulatory elements. Therefore, further studies are required to determine the occupancy of their independent regulatory mechanism and mutually inhibitory mechanism on VMP1 and miR-21 expression.

More importantly, this study demonstrated that TFEB is involved in the miR-21/VMP1 feedback loop and miR-21 could regulate autophagy through TFEB in CRC cells. TFEB, a master transcription factor for autophagosome formation and lysosomal biogenesis, coordinated the transcriptional program that controls autophagic process by driving autophagic and lysosomal genes expression, such as LC3, WIPI, ATG9, LAMP1^37^. Whether TFEB regulates VMP1 expression? Our results indicated TFEB activates VMP1 transcription by directly binding to the promoter region of VMP1, and TFEB-induced upregulation of VMP1 transcription inhibited miR-21 expression, as did saRNA-158 or GLI3. In addition, we revealed that miR-21 inhibited TFEB activity and nucleus translocation via PTEN/AKT, by which miR-21 inhibited the autophagy activity in CRC cells. It is known that PTEN is a target gene of miR-21^7^, and can inhibit the activation of PI3-K/AKT pathway^38^. Previous studies have revealed that AKT directly mediated TFEB phosphorylation at Ser467 and prevented its nuclear translocation, by which AKT can inhibit autophagy-lysosomal pathway^32,39^. In addition, AKT also inhibited autophagy occurrence via mTORC1^40^. In this study, knock-down of miR-21 significantly increased TFEB nuclear translocation through enhancing the inhibitory of PTEN on AKT phosphorylation, leading to increasing autophagic genes expression and upregulation of autophagy activity, indicating that the oncogene miR-21 is involved in regulating autophagy in CRC cells. It is well known that autophagy is not only a self-protection mechanism of cells, but also a programmed cell death mechanism^41–44^. Therefore, there may be a link between miR-21 functions as an “oncomiR” and its regulation of autophagy activity in CRC cells.

It’s known that 5-FU-based chemotherapy is widely used in CRC, but the prognosis of patient with malignant CRC is poor due to the primary or acquired resistance to 5-FU^25,45,46^. In our study, we found that miR-21 was significantly increased, adversely, VMP1 was lower expressed in HCT116-FUR cells compared to parental HCT116 cells. Previous studies also have shown that miR-21 plays an important role in drug resistance in CRC cells, and miR-21 overexpression dramatically reduced the therapeutic efficacy of 5-FU both in vitro and in vivo^47–49^. In addition, VMP1 is also an essential protein involved in autophagy, cell proliferation, invasion, and metastatic potential of cancer cells^19,22,23^. Downregulation of VMP1 increased the malignancy of various tumors, including CRC^21–23,50^. The regulation loop of miR-21/VMP1 can increase miR-21 expression and reduce VMP1 expression, which could reduce the drug sensitivity of tumor cells to 5-FU. Our results showed that breaking this feedback loop could decrease the expression of miR-21, significantly reduce the migration and invasion ability of HCT116 cells, and also increase the drug sensitivity to 5-FU. In addition, mounting evidence suggests that miR-21 expression is predominantly upregulated in the stromal fibroblastic cells in CRC tumors and promotes the spread, invasiveness, and chemoresistance in neighboring CRC cells via paracrine signaling^51–53^. Therefore, we hypothesized that the high expression of miR-21 in the stromal fibroblastic cells in CRC tumors could inhibit VMP1 transcription, which in turn increases miR-21 expression. Thus miR-21 would increase itself expression via miR-21/VMP1 feedback loop in the tumor microenvironment of CRC. Disrupting the miR-21/VMP1 feedback loop will decrease miR-21 expression, and reduce the malignancy of CRC tumors.

Collectively, our study revealed a novel feedback loop of miR-21/VMP1 regulating miR-21 expression. Meanwhile, we demonstrated the role of this feedback loop on tumor metastasis, invasion, proliferation, and multidrug resistance in CRC cells. Thus, our study provided a new theoretical clue for the targeted therapy of miR-21 to reduce the malignancy of CRC, including enhancing drug sensitivity and inhibiting metastasis.

## Materials and methods

### Cell Culture

The human CRC lines HCT116, RKO, LOVO, DLD1, Caco-2, HT-29, SW480 were obtained from the Cell Bank of the Chinese Academy of Sciences (Shanghai, China). RKO-miR-21-KO cell was a kind gift from Dr. Lin Zhang at the University of Pittsburgh. Cells were cultured with DMEM high glucose medium (Hyclone) containing 10% FBS (BI) and 1% penicillin/streptomycin (Gibco). The HCT116-FUR cell line was established by exposure to increasing concentrations of 5-FU. Briefly, HCT116 cells were initially cultured in DMEM containing 5-FU at the concentration of 5 µM for 2 weeks, and then cultured in the medium without 5-FU for 2 weeks. The survival cells were treated with a higher concentration of 5-FU in DMEM. 5-FU concentration doubled every 4 weeks. Finally, the resultant cells with exponential growth under high concentration of 5-FU were considered as 5-FU resistant cells, and named HCT116-FUR.

### RNA oligos, plasmids, and antibodies

PTEN siRNA (siPTEN-1: 5′-GGUGUAAUGAUAUGUGCAUTT; siPTEN-2: 5′-GCUACCUGUUAAAGAAUCATT; siPTEN-3: 5′-CGGGAAGACAAGUUCAUGUTT); TFEB siRNA (siTFEB-1: 5′-CAGGCUGUCAUGCAUUACATT; siTFEB-2: 5′-GGCUACAUCAAUCCUGAAATT; siTFEB-3: 5′-GACGAAGGUUCAACAUCAATT) were designed according to the cDNA sequence. The mimic has-miR-21 (5′-UAGCUUAUCAGACUGAUGUUGA), VMP1 saRNA (saRNA-158: 5′-CUACUGGCGAGAAAAUGAATT; saRNA-227: 5′-GGCGGAAGACAGUUUAUAATT; saRNA-328: 5′-GCUGGGAACUAGAAAUUCUTT; saRNA-479: 5′-CUAUAUUCUUAGAAACCAATT; saRNA-830: 5′-GUUAAUCUCUUUGUGUGAATT), and the negative control RNA oligo (UUCUUCGAACGUGUCACGUTT) were synthesized by GenePharam (Shanghai, China). VMP1 cDNA was amplified from total cDNA of HCT116 cells, then cloned into pcDNA3.1(+) (pcDNA3.1-VMP1) and cloned into pLenti (pLenti-VMP1) by a *Eco*RI and *Xho*I enzymes. pEGFP-C3-hGli3 and pCIP-caTfeb vectors were obtained from addgene. The primers for cloning VMP1 are listed as follows: *Eco*RI-VMP1-F: CCGGAATTCATGGCAGAGAATGGAAAAAATTG; *Xho*I-VMP1-R: CCGCTCGAGATTTAGTTTTCTCCTCTGAGTTC. The anti-VMP1 rabbit monoclonal antibody (#12929), anti-TFEB rabbit monoclonal antibody (#37785) and anti-phospho-STAT3 (Tyr705) rabbit monoclonal antibody (#9145) were purchased from Cell Signaling Technology (CST, USA). The anti-LC3 rabbit polyclonal (306019), anti-β-Tubulin mouse monoclonal (200608), anti-Histone H3 rabbit polyclonal (381572), anti-GAPDH mouse monoclonal (200306) antibodies were purchased from Zen BioScience (China). The anti-PTEN mouse monoclonal (CY5231), anti-pan-AKT rabbit monoclonal (CY5561), anti-phospho-AKT (Ser473) rabbit polyclonal (CY6569) antibodies were purchased from Abways (China). The anti-Actin rabbit polyclonal (20536) antibody was purchased from Proteintech (USA).

### Transfection

HCT116 and RKO cells were transfected with 100pmol siRNA/saRNA or 5 μg plasmid each well for certain time points using Lipofectamine 3000 Transfection Reagent (Invitrogen, Carlsbad, CA, USA) according to the manufacturer’s instructions. Cells were collected 48 h after transfection for RNA or protein analysis.

### Real-time quantitative PCR

The total RNA of cells and tissues was extracted using the RNAiso Plus (Takara, Tokyo, Japan) according to the manufacturer’s instructions. cDNA synthesis was carried out using HiScriptTM Q-RT Super Mix for qPCR (+gDNA wiper) (Vazyme biothech, China). Real-time PCR was carried out on BIO-RAD CFX96 (Bio-Rad Laboratories) using SYBR Green Master Mix (Vazyme biothech, China). Primers for qPCR are listed as follows: GAPDH (F: TGCACCACCAACTGCTTAGC; R: GGCATGGACTGTGGTCATGAG), VMP1 (F: GACCAGAGACGTGTAGCAATG; R: ACAATGCTTTGACGATGCCATA), TFEB (F: ACCTGTCCGAGACCTATGGG; R: CGTCCAGACGCATAATGTTGTC), STAT3 (F: ATCACGCCTTCTACAGACTGC; R: CATCCTGGAGATTCTCTACCACT) GLI3 (F: TGGTTACATGGAGCCCCACTA; R: GAATCGGAGATGGATCGTAATGG), miR-21(miR-21-RT: GTCGTATCCAGTGCAGGGTCCGAGGTATTCGCACTGGATACGACTCAACAT. miR-21 F: ATGGGCTGTCTGACATTTTGGTA; miR-21 R: CATTGGATATGGATGGTCAGATGA. VMP1-miR-21 F: GCAAGCACATAGTGGAGCAAAT; VMP1-miR-21 R: CCGTTGAGCCTCCAGGTACTC. pri-miR-21 F: AGCCTGTTAGCTACAGTTGC; pri-miR-21 R: CTCCAGTACATTAGTAACAGC), U6 (U6-RT: CGCTTCACGAATTTGCGTGTC; U6 F: TCGCTTCGGCAGCACATATAC; U6 R: GCGTGTCATCCTTGCGCAG). Four primary CRC tissue samples and paired adjacent non-cancer tissues used for qPCR analysis were provided by Prof. Yuanyuan Lu at Xijing Hospital of Digestive Diseases.

### Immunohistochemistry (IHC) analysis

IHC was carried out to analyze the altered protein expression of VMP1 in human CRC tissues and paired adjacent non-cancer tissues. The primary antibody against VMP1 (#D262380, Sangon Biotech) for IHC with 1:50 dilution. For quantitative analyses, the IHC images were subjected to integrated optical density (IOD) measurements using Image-Pro Plus 6.0 to calculate the average optical density (AOD) using the formula: AOD = IOD/Area. Four primary CRC tissue samples and paired adjacent non-cancer tissues used for IHC analysis were provided by Dan Jiang at West China Hospital of Pathology, Sichuan University.

### Cell Proliferation and Viability Assays

Cells were seeded in 96-well micro-titer plates at an initial density of 10000 cells/well. After incubation for 24 h, cells were incubated with serial dilution of 5-FU for 48 h. Then 10 μl of MTT solution (1 mg/ml) was added to each well. After incubation for 4 h, the supernatant was aspirated and 100 μl DMSO were added into each well. Absorbance was measured on a microculture plate reader at 490 nm. Data represent mean ± SD from three independent experiments.

### Colony formation assay

Cells were trypsinized by 0.25% trypsin and counted. 800 cells were seeded in the 6-well plate and incubated in the CO_2_ incubator. After 2 weeks, colonies were stained by 0.5% crystal velvet and the number of colonies was counted under microscopy.

### Dual-luciferase reporter assay

VMP1 promoter (−1000/+100) and pri-miR-21 promoter (−1000/+100) were synthesized by TSINGKE, and was used to replace the PGK promoter in pmiRGLO vector. HCT116 and RKO were transfected with pCIP-caTfeb vector and the construct dual-luciferase vectors (pmiRGLO-VMP1 promoter and pmiRGLO-pri-miR-21 promoter), 48 h post-transfection, cells were collected to detect the dual-luciferase activity according to the manufacturer’s instructions (Dual-Luciferase Reporter Assay System, #E1910, Promega). The VMP1 promoter-Luc activity was normalized to Rluc activity.

### Chromatin immunoprecipitation (CHIP) assay

CHIP assay was conducted following the CHIP kit protocol (#9005, CST). The primers used for VMP1 promoter are listed as follows: F: CTGTAAGGAGCAACCAACCT; R: TCAGTTAGGCTCTTAGCGG. The primers used for pri-miR-21 promoter are listed as follows: F: GGATGACACAAGCATAAGTCA; R: GATACTGTACTCTGGTATGGC.

### Transwell assay

The experiment was conducted using 24-well transwell chambers (#353097, FALCON) with 100 μl of cold coating buffer containing 200 μg/ml of Matrigel (#356230, Corning). Cells (2.5 × 10^4^) were initially suspended with 400 μl of basic medium into the upper chamber, and 600 μl of complete medium containing 15% FBS was added into the lower chamber. The chamber was taken out after incubated for 48 h, then fixed and stained to count the number of cells passing through the chambers.

### Statistical analysis

Statistical analyses were performed using GraphPad Prism 7.02. Continuous data were presented as the mean ±standard deviation (SD) for at least 3 repeated individual experiments for each group. Statistical differences were determined by using ANOVA and Student’s *t* test for independent samples. A value of *P* < 0.05 was considered statistically significant.

## Supplementary information

Supplementary figure legends

Supplementary Figure1

Supplementary Figure2

Supplementary Figure3
