# Supplementary material for: An autoregulatory feedback loop of miR-21/VMP1 is responsible for the abnormal expression of miR-21 in colorectal cancer cells
Source: Cell Death Dis. 2020 Dec 14;11(12):1067. doi: 10.1038/s41419-020-03265-4 (PMC7736343; doi:10.1038/s41419-020-03265-4)
Supplement: Supplementary file 1 — Supplementary figure legends [file 41419_2020_3265_MOESM1_ESM.pdf]

Supplementary Figure legends

Supplementary Figure 1. A. The mRNA expression level of miR-21 in human colorectal cancer was analyzed using the Oncomine TCGA Colorectal dataset. The mRNA expression level of VMP1 in human colorectal cancer were analyzed using the Oncomine Skrzypczak Colorectal dataset. **B.** MTT assay to calculate the concentration of 5-FU resistance achieved in case of HCT116-FUR cells. Date are presented as mean±SD. ***P<0.001.

Supplementary Figure 2. A. The primer binding sites of VMP1 and miR-21 transcripts. RT-PCR primers are depicted as facing arrows, reverse transcription primer is depicted as single thick arrow. **B.** HCT116 was starved with glucose-free medium for 2h and 4h, the expression of miR-21, and the amount of pri-miR-21 and VMP1-miR-21 transcripts, were measured by qPCR. The protein expression level of VMP1 was measured by Western blot. **C.** HCT116 was treated with 5μM rapamycin for 12h, 24h and 48h, the expression of miR-21, and the amount of the pri-miR-21 and VMP1-miR-21 transcripts, were measured by qPCR. The protein expression level of VMP1 was measured by Western blot. Date are presented as mean ±SD. ***P<0.001, **P<0.01, *P<0.05.

Supplementary Figure 3. A. RKO and HCT116 were transfected with miR-21 nucleotide mimic, and cells were treated with 1μM Afuresertib after transfection for 24h, the protein level of TFEB in cytoplasm and nucleus respectively was measured by Western blot, H3 and β-actin were used as nuclear and cytosolic markers, respectively. **B.** The protein expression level of TFEB and VMP1 were measured by Western blot after over-expression of the constitutively active TFEB by pCIP-caTfeb vector or knock-down of TFEB

expression by siRNA in HCT116 cells. **C.** The predicted binding sites of AP-1 and AR on VMP1 promoter region were shown.
