# Supplementary figures and images for: An autoregulatory feedback loop of miR-21/VMP1 is responsible for the abnormal expression of miR-21 in colorectal cancer cells
Source: Cell Death Dis. 2020 Dec 14;11(12):1067. doi: 10.1038/s41419-020-03265-4 (PMC7736343; doi:10.1038/s41419-020-03265-4)

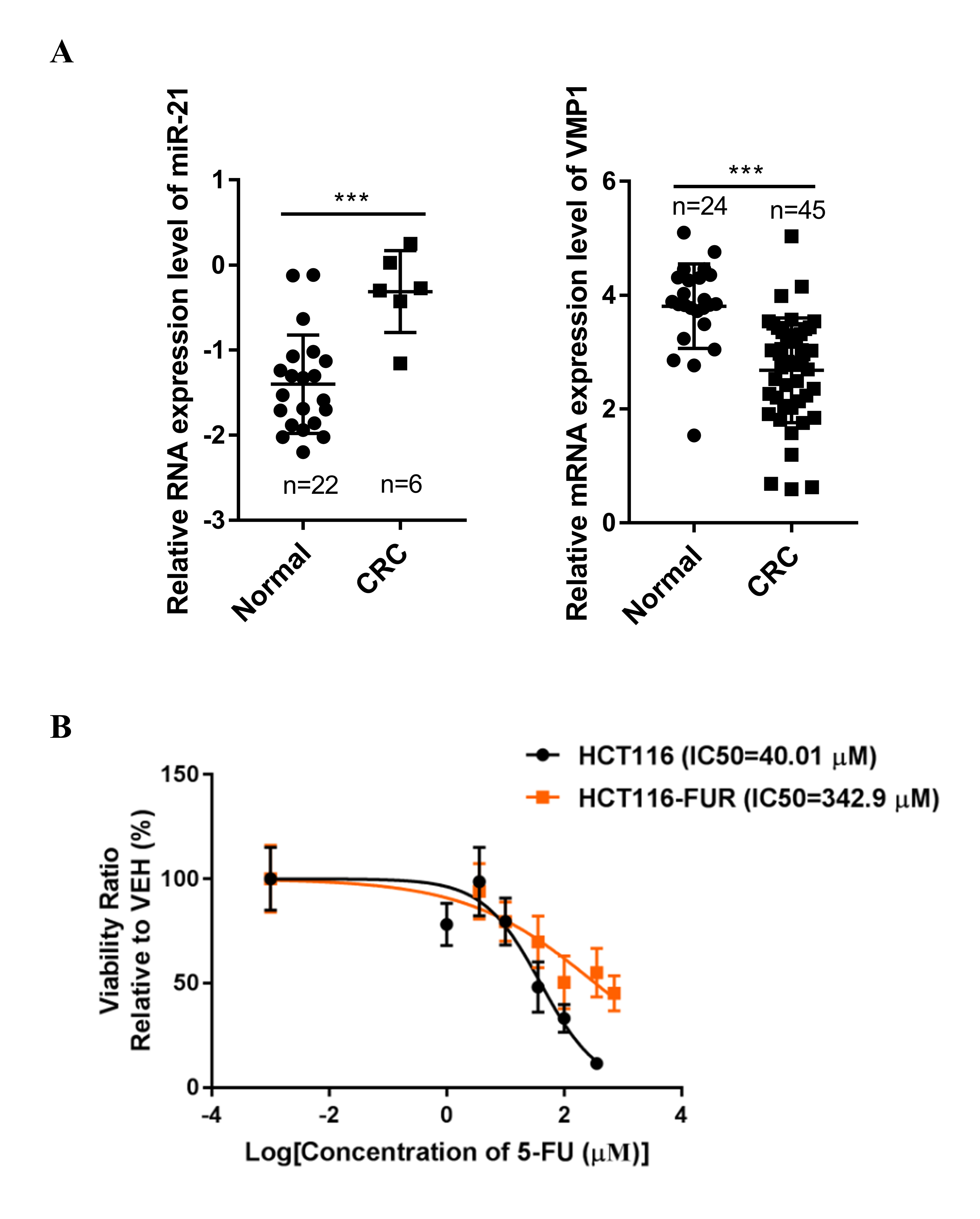

Supplement: Supplementary file 2 — Supplementary Figure1 [file 41419_2020_3265_MOESM2_ESM.tif]

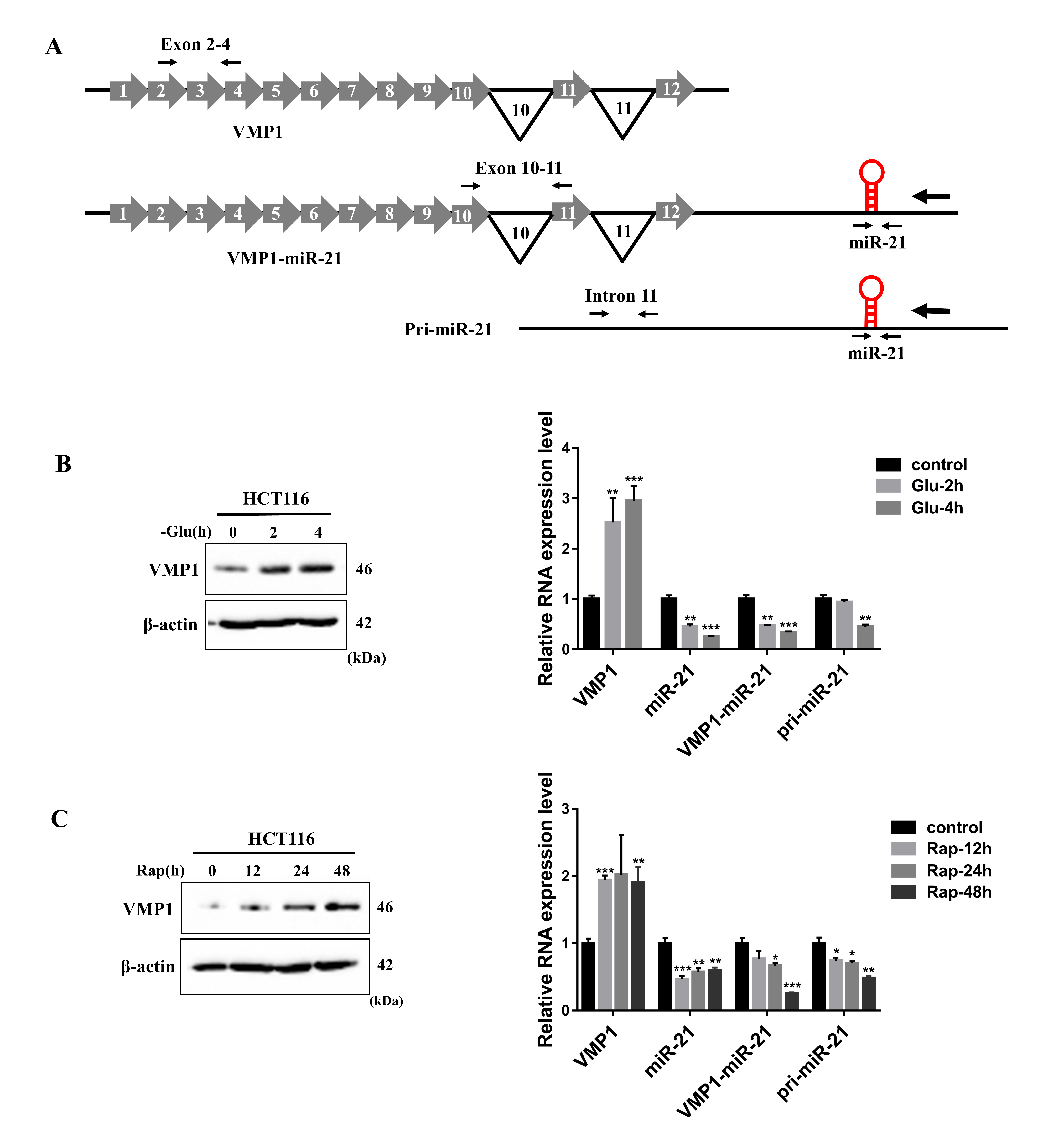

Supplement: Supplementary file 3 — Supplementary Figure2 [file 41419_2020_3265_MOESM3_ESM.tif]
